# SIRT7 safeguards ERα proteostasis *via* deacetylation-dependent degradation of unliganded and misfolded receptors

**DOI:** 10.1016/j.jbc.2026.111173

**Published:** 2026-01-20

**Authors:** Mengdi Cao, Junfeng Zhang, Huixia Liu, Ronghui Fan, Faliang Wu, Yitong Meng, Tiansheng Li, Yalan Wu, Xiaolong Tang

**Affiliations:** 1 Hunan Key Laboratory of Animal Models and Molecular Medicine, School of Biomedical Sciences, Hunan University, Changsha, China; 2 Department of Histology and Embryology, School of Basic Medical Sciences, Xiangya School of Medicine, Central South University, Changsha, China

**Keywords:** SIRT7, protein degradation, ERα, estrogen signaling, receptor quality control

## Abstract

SIRT7 has been implicated in diverse physiological and pathological processes, yet its role in sexual dimorphism and the underlying molecular mechanisms remains insufficiently explored. Given that ERα-mediated estrogen signaling is a central regulator of sexual dimorphism and that ERα undergoes stringent quality control to preserve signaling sensitivity, we investigated whether SIRT7 and ERα are mechanistically connected. Here, we identify SIRT7 as a molecular inspector that safeguards the quality of estrogen receptor α (ERα) to fine-tune estrogen signaling through the regulation of ERα proteostasis. Under estrogen-deprived conditions or in the presence of misfolded ERα, SIRT7 deacetylates ERα and promotes its degradation through the E3 ubiquitin ligase STUB1, thereby maintaining a functional receptor pool and preserving estrogen responsiveness. During this process, deacetylated ERα competes with SIRT7 for STUB1 binding, an E3 ligase that is also required for SIRT7 protein turnover, thus leading to SIRT7 stabilization. As a feedback mechanism, upon estrogen (E2) stimulation, E2-bound ERα activates non-genomic MAPK signaling to trigger SIRT7 degradation *via* another E3 ligase UBR5, which ensures the proper receptor signaling activation. Given the central role of ERα in aging and hormone-related cancers, our findings highlight SIRT7 as a key regulator linking age-associated disorders and hormone-driven tumorigenesis.

SIRT7, the last identified member of the sirtuin family, is primarily localized in the nucleolus but possesses the ability to shuttle between the nucleus and cytoplasm (1, 2, 3). Emerging evidence reveals that SIRT7 plays critical roles across multiple tissues and organs, and its dysregulation participates in diverse physiological and pathological processes (4). For instance, SIRT7 has been reported to suppress endoplasmic reticulum (ER) stress and protect against fatty liver disease (5), regulate hepatic lipid metabolism through the ubiquitin-proteasome pathway (6), and modulate metabolic and inflammatory gene expression during senescence and liver aging (7). Intriguingly, discrepancies have been observed. In some reports, Sirt7 knockout mice are resistant to high-fat diet-induced hepatic steatosis, obesity, and glucose intolerance, with reduced hepatic triglyceride accumulation (6). In contrast, other studies show that SIRT7-deficient mice spontaneously develop chronic hepatosteatosis resembling human fatty liver disease (5). Indeed, the functions of SIRT7 in liver cancer are likewise diverse and, to some extent, contradictory. Early studies described SIRT7 as an oncogenic factor in human hepatocellular carcinoma (8), whereas subsequent work demonstrated that SIRT7 deficiency promotes HCC progression in mouse models (9). Moreover, similar discrepancies have been reported in breast cancer: while some studies suggest that SIRT7 suppresses breast cancer metastasis (10), others indicate that SIRT7 activity is required for tumor progression (11). It is notable that those tissues in which SIRT7 exerts context-dependent effects, such as the mentioned liver and mammary gland, possess pronounced sexual dimorphism. The liver, for example, displays sex-specific differences in structure, metabolic function, and disease susceptibility (12), while mammary tissue is well known to be regulated by estrogen (13). Thus, although variations in genetic background or experimental design may account for some inconsistencies, the potential involvement of SIRT7 in regulating sexual dimorphism warrants further investigation to address the possibility of hormonal context functions.

Estrogens are well known to regulate sexual dimorphism in physiological and pathological conditions, among which 17β-estradiol (E2) is the predominant and most biologically active form in premenopausal women (14). The actions of estrogen are mediated by the estrogen receptors ERα and ERβ, which act as ligand-activated transcription factors (15). Previous studies have pointed that these sexual dimorphism effects are primarily mediated through ERα (16). Genetic ablation of ERα in mice majorly accounts for the phenotypical changes in obesity, insulin resistance, and glucose intolerance (17). Upon estrogen binding, the ligand-binding domain (LBD) of ERα undergoes a conformational change that promotes receptor dimerization, DNA binding, and subsequent transcriptional activation of target genes (18). The cellular response to estrogen is tightly regulated, and a large repertoire of ERα-interacting proteins has been identified as coactivators or corepressors that modulate its transcriptional activity (19). Interestingly, the native hormone-free state of ERα is unstable and dynamic, which requires a large multiprotein complex to maintain its stability, such as the chaperone protein Hsp70 and Hsp90 (20). Misfolded receptors lead to reduced estrogen responsiveness and compromised estrogen signaling, as observed during aging or tumorigenesis. However, the mechanisms governing ERα protein regulation, particularly in sexually dimorphic tissues and organs, remain largely unexplored.

In this study, we uncover an unexpected mutual regulation between SIRT7 and estrogen signaling, which is governed by ERα under different ligand-bound states. We demonstrate that, upon estrogen exposure, E2-bound ERα triggers SIRT7 degradation, whereas under ligand-free conditions or stress-induced misfolded conformation, ERα is deacetylated by SIRT7 and undergoes protein degradation.

## Results

### E2 triggers SIRT7 protein degradation

**Figure 1:**
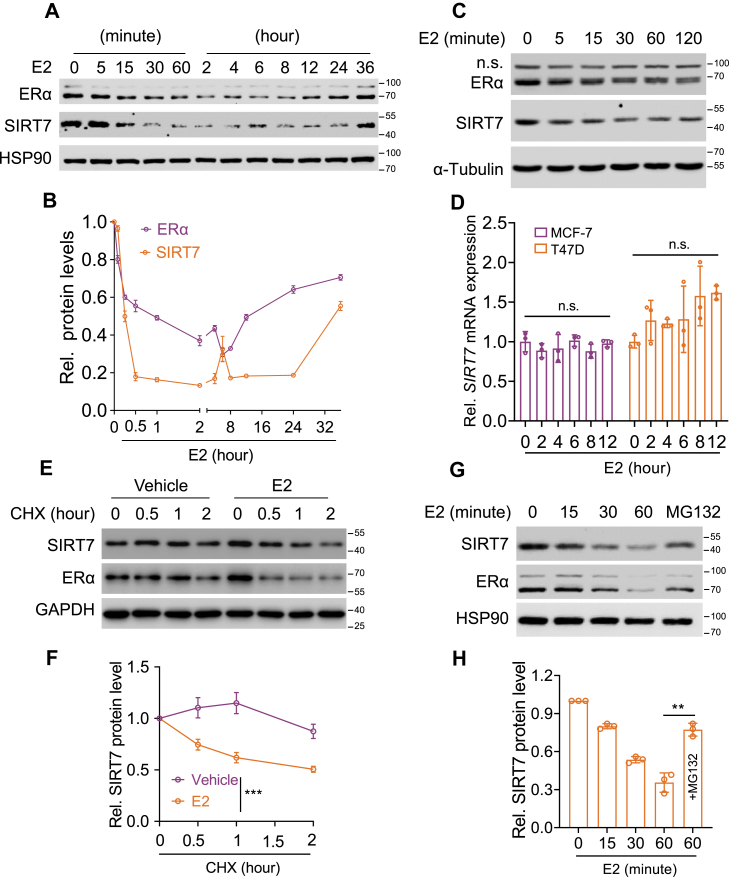
**E2 facilitates SIRT7 protein degradation.***A–B*, Immunoblots (*A*) showing expression of SIRT7 and ERα in MCF-7 cells following incubation with E2 for the indicated time points, with quantification in (*B*) presenting as mean ± SD from three independent experiments. *C*, immunoblots showing the expression of ERα and SIRT7 in ERα-positive T47D cells treated with E2 for the indicated time points. *D*, quantitative PCR (qPCR) analysis of *SIRT7* mRNA expression in both T47D and MCF-7 cells exposed to E2 for the indicated time points; Bars, mean ± SD from three independent experiments; The *p* value, unpaired two-sided Student’s *t* test; n.s., no significance. *E–F*, immunoblotting (*E*) analysis of SIRT7 protein stability in MCF-7 cells treated with E2 and cycloheximide (CHX, 50 μg/ml), with protein levels quantified in (*F*) as mean ± SD from three independent experiments; The *p* value calculated by two-way ANOVA, ∗∗∗*p* < 0.001. *G–H*, immunoblots (*G*) showing SIRT7 protein expression in MCF-7 cells treated with E2 and/or MG132 (10 μM) for the indicated time points, with quantification in (*H*) representing mean ± SD from three independent experiments; The *p* value determined by unpaired two-sided Student’s *t* test, ∗∗*p* < 0.01.

To determine whether SIRT7 is involved in estrogen signaling, we first examined the effect of 17β-estradiol (E2) on SIRT7 expression in ERα-positive MCF-7 cells. Initially, E2 treatment markedly reduced ERα protein level (Fig. 1*A*), consistent with the notion of E2-induced ERα degradation during receptor activation (21). Interestingly, this process accompanied by a pronounced decrease in SIRT7 protein (Fig. 1*B*). Further, the decline in SIRT7 protein occurred as early as 30 min after E2 treatment in both MCF-7 (Fig. 1*A*) and T47D (another ERα-positive breast cancer cell line, Fig. 1*C*) cells, but their mRNA expression levels were marginally changed (Fig. 1*D*), suggesting the influence on protein stability. We thus performed the cycloheximide (CHX) chase assay and found that E2 compromised SIRT7 protein stability (Fig. 1, *E* and *F*). Of great note, such effect was apparently prevented by the addition of MG132 (Fig. 1, *G* and *H*), implicating the proteasome-dependent manner. These results therefore suggest a potential link between SIRT7 and E2 signaling.

### Unliganded-ERα stabilizes SIRT7 protein

**Figure 2:**
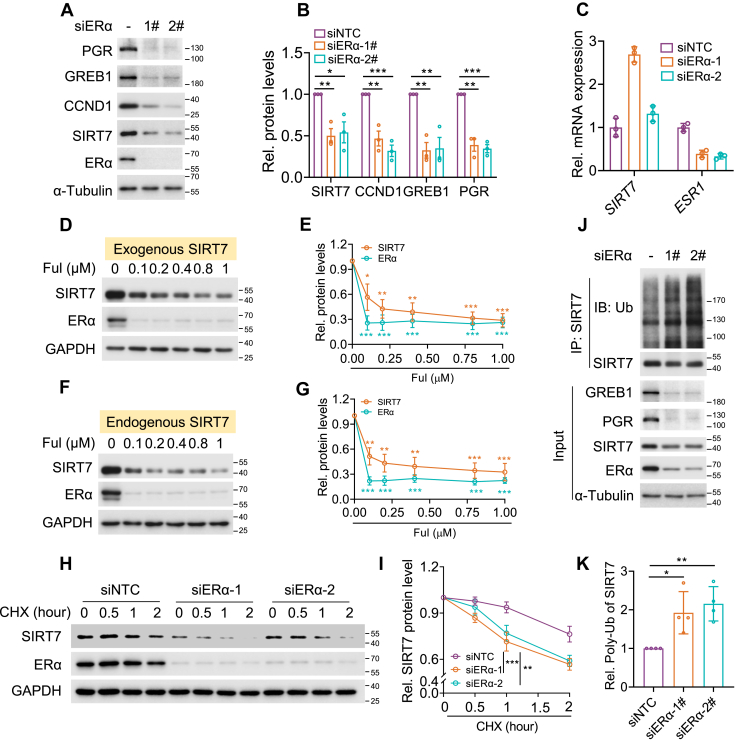
**Unliganded-ERα stabilizes SIRT7 protein.***A****–****B*, Immunoblotting (*A*) analysis and related quantification (*B*) showing the expression changes of SIRT7 and ERα downstream targets in MCF-7 cells following ERα knockdown; Bars represent mean ± SD, with the *p* values calculated by unpaired two-sided Student’s *t* test, ∗*p* < 0.05, ∗∗*p* < 0.01, ∗∗∗*p* < 0.001. *C*, qPCR analysis of *SIRT7* and *ERα* mRNA levels in MCF-7 cells subject to knockdown of ERα; Bars, mean ± SD. *D–G*, immunoblots (*D* and *F*) showing exogenous (*D*) or endogenous SIRT7 (*E*) protein levels in MCF-7 cells treated with various dose of fulvestrant (Ful) for 12 h, with quantification in (*E* and *G*) presented as mean ± SD; The *p* values determined by unpaired two-sided Student’s *t* test, ∗*p* < 0.05, ∗∗*p* < 0.01, ∗∗∗*p* < 0.001. *H–I*, immunoblotting (*H*) analysis and protein quantification (*I*) of SIRT7 protein turnover in MCF-7 cells treated with E2, in the presence of ERα knockdown or not; Curves, mean ± SD, from three independent experiments; The *p* values determined by two-way ANOVA, ∗∗*p* < 0.01, ∗∗∗*p* < 0.001. *J–K*, Immunoblots (*J*) and related quantification (*K*) showing the levels of ubiquitinated SIRT7 in MCF-7 cells with or without ERα knockdown under ligand free condition; Bars, mean ± SD; The *p* values by unpaired two-sided Student’s *t* test, ∗*p* < 0.05, ∗∗*p* < 0.01.

Given that ERα is one of the principal receptors mediating E2 signaling, we next examined its impact on SIRT7 protein stability. Intriguingly, under estrogen-deprived conditions, ERα knockdown markedly reduced SIRT7 protein abundance (Fig. 2, *A* and *B*), without downregulating its mRNA expression (Fig. 2*C*). Likewise, treatment with fulvestrant (Ful), a selective estrogen receptor degrader (SERD) that promotes ERα protein degradation, effectively diminished the protein levels of both endogenous and exogenous SIRT7 (Fig. 2, *D*–*G*). Moreover, employing CHX chase assays revealed that ERα loss significantly accelerated SIRT7 protein turnover (Fig. 2, *H* and *I*), and facilitated its polyubiquitination under ligand-free conditions (Fig. 2, *J* and *K*). These findings indicate that unliganded ERα, in contrast to E2-induced protein degradation, exerts a protective effect on SIRT7 protein stability.

### E2-bound ERα promotes SIRT7 degradation *via* UBR5

**Figure 3:**
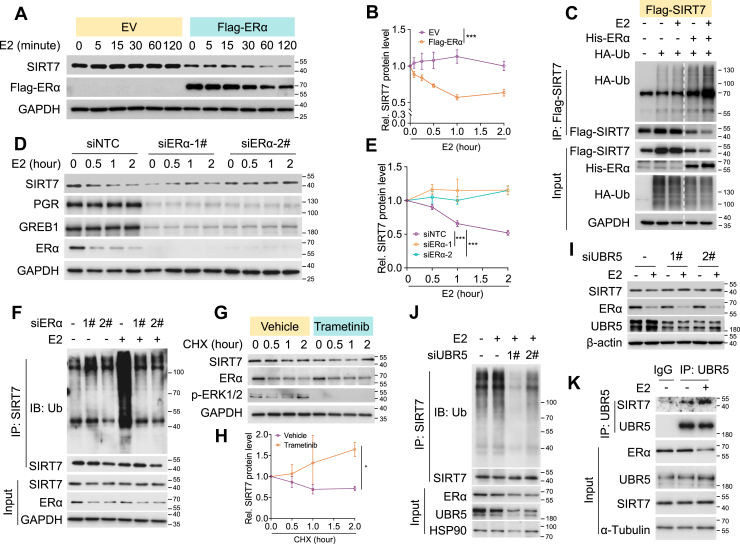
**E2-bound ERα promotes SIRT7 degradation *via* UBR5.***A–B*, immunoblotting (*A*) and related quantification (*B*) analysis of SIRT7 expression in MDA-MB-231 cells with or without exogenous ERα restoration (EV: empty vector), following E2 exposure for the indicated durations. Curves, mean ± SD from three independent experiments; The *p* value calculated by two-way ANOVA, ∗∗∗*p* < 0.001. *C*, immunoblots showing the ubiquitinated SIRT7 in MDA-MB-231 cells transfected with the indicated plasmids. *D–E*, immunoblotting (*D*) and related quantification (*E*) analysis of SIRT7 protein turnover in MCF-7 cells treated with E2, with or without ERα knockdown. Curves, mean ± SD from three independent experiments. The *p* value determined by two-way ANOVA, ∗∗∗*p* < 0.001. *F*, immunoblotting analysis of SIRT7 polyubiquitination in MCF-7 cells treated with E2, with or without ERα knockdown. *G–H*, immunoblotting (*G*) and protein quantification (*H*) analysis of SIRT7 expression in MCF-7 cells under the exposure of E2 and with or without treatment by ERK1/2 inhibitor trametinib (25 nM). Curves, mean ± SD from three independent experiments; The *p* value calculated by two-way ANOVA, ∗*p* < 0.05. *I*, immunoblotting analysis of SIRT7 expression in MCF-7 cells treated with E2 and with or without UBR5 knockdown. *J*, Immunoblot showing the ubiquitinated SIRT7 in MCF-7 cells with UBR5 knockdown and/or E2 exposure. *K*, immunoblots showing the interaction between UBR5 and SIRT7 in MCF-7 cells treated with E2 or not.

Because E2 promoted SIRT7 protein degradation whereas unliganded ERα stabilized it, we next examined whether this effect of E2 was determined by liganded ERα. In ERα-negative MDA-MB-231 cells, E2 treatment had little effect on SIRT7 protein expression (Fig. 3, *A* and *B*, EV). However, once with re-expression of ERα, E2 achieved apparent potency to promote SIRT7 protein reduction (Fig. 3, *A* and *B*, Flag-ERα). Accordingly, E2 significantly enhanced SIRT7 polyubiquitination only in MDA-MB-231 cells expressing ectopic ERα, but not in controlled cells (Fig. 3*C*). Furthermore, in MCF-7 cells expressing endogenous ERα, ERα depletion markedly attenuated E2-induced SIRT7 protein degradation as evaluated by CHX assay (Fig. 3, *D* and *E*). In agreement, E2 substantially increased SIRT7 polyubiquitination, while this effect was largely diminished by ERα knockdown (Fig. 3*F*). E2-liganded ERα is known to activate non-genomic estrogen signaling, such as the MAPK/ERK pathway (22). Interestingly, such ERK activation is reported to engage UBR5-mediated SIRT7 degradation (23). We therefore inferred that E2-induced SIRT7 degradation was driven by ERK1/2 activation and the E3 ligase UBR5. Indeed, treatment with the ERK1/2 inhibitor trametinib (ERKi) markedly prevented E2-triggered SIRT7 protein downregulation (Fig. 3, *G* and *H*). Similarly, UBR5 knockdown remarkably restored SIRT7 protein level in E2-treated cells (Fig. 3*I*) and substantially reduced E2-enhanced SIRT7 polyubiquitination (Fig. 3*J*). Supporting this mechanism, E2 significantly strengthened the interaction between SIRT7 and UBR5 (Fig. 3*K*). Collectively, these results demonstrate that E2-liganded ERα promotes SIRT7 proteasomal degradation through the E3 ubiquitin ligase UBR5.

### STUB1 is essential for the degradation of both ERα and SIRT7 under ligand-free statuses

**Figure 4:**
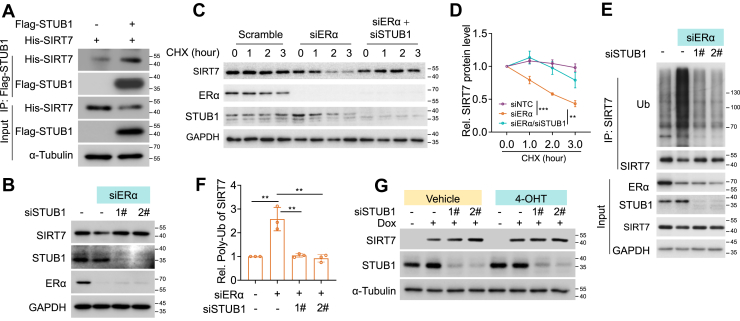
**STUB1 is essential for both ERα and SIRT7 degradation under ligand-free status.***A*, immunoblotting detecting the interaction of STUB1 and SIRT7 in the anti-Flag- STUB1 co-immunoprecipitation elutes from HEK293 cells with indicated transfections. *B*, immunoblotting analysis of SIRT7 expression in MCF-7 cells cultured in E2-free medium, following ERα or/and STUB1 knockdown. *C–D*, immunoblots (*C*) and indicated quantification (*D*) showing CHX chase assays for SIRT7 in MCF-7 cells cultured in E2-deprived medium, with or without ERα and/or *STUB1* knockdown. Curves, mean ± SD from three independent experiments; The *p* value calculated by two-way ANOVA, ∗∗*p* < 0.01, ∗∗∗*p* < 0.001. *E–F*, immunoblots (*E*) and protein quantification (*F*) showing the ubiquitinated SIRT7 in MCF-7 cells as treated in (*C*). Bars, mean ± SD from three independent experiments; The *p* value by unpaired two-sided Student’s *t* test, ∗∗*p* < 0.01. *G*, immunoblotting analysis of SIRT7 protein expression in MCF-7 cells cultured in E2-free medium, following 4-OHT treatment or *STUB1* knockdown.

In the absence of ligand, ERα forms a multiprotein complex with molecular chaperones such as Hsp70 and Hsp90 to maintain receptor stability. Notably, STUB1, an E3 ubiquitin ligase associated with the Hsp70 complex, has been shown to mediate ERα protein degradation (24). Interestingly, we found that SIRT7 co-immunoprecipitated with STUB1 (Fig. 4*A*), and that silencing STUB1 restored SIRT7 protein expression in ERα-depleted MCF-7 cells under ligand-free conditions (Fig. 4*B*). Furthermore, STUB1 knockdown markedly attenuated the ERα loss-induced SIRT7 protein degradation (Fig. 4, *C* and *D*) and polyubiquitination (Fig. 4, *E* and *F*). In addition, STUB1 depletion primarily increased SIRT7 protein level under ligand-free conditions, whereas treatment with 4-hydroxytamoxifen (4-OHT), which stabilizes ERα, had minimal effect (Fig. 4*G*). Collectively, these results indicate that STUB1 mediates SIRT7 protein degradation in the absence of ERα under ligand-free conditions.

### Deacetylation of ERα at K302/303 promotes SIRT7 stabilization and modulates its response to E2

**Figure 5:**
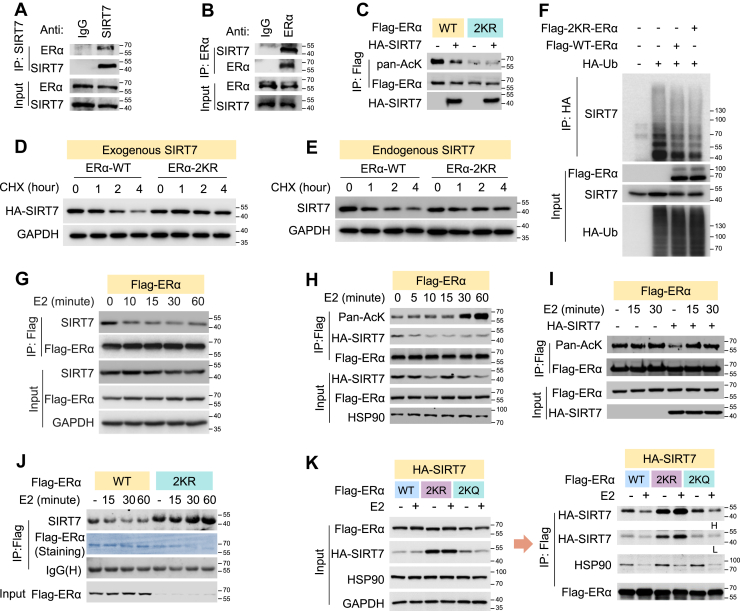
**Deacetylation of ERα at K302/303 leads to SIRT7 stabilization and a distinct response to E2.***A–B*, immunoblots showing the protein interaction between SIRT7 and ERα in MCF-7 cells by co-immunoprecipitation (Co-IP) assays. *C*, immunoblots showing the acetylation level of WT-ERα and K302/303R mutant (2KR) under SIRT7 overexpression or not. *D–E*, immunoblots showing CHX chase assays for SIRT7 in HEK293 cells cultured in E2-deprived medium and with transfection of ectopic WT-ERα and 2KR mutant, respectively. *F*, immunoblotting analysis of ubiquitinated SIRT7 in HEK293 cells with transfection of indicated plasmids. *G*, immunoblots showing the interaction between ERα and SIRT7 in HEK293 cells treated with E2 for indicated time points. *H*, immunoblotting analysis of ERα acetylation levels in HEK293 cells transfected with the indicated plasmids and treated with E2 for various time points. *I*, immunoblots showing the acetylated ERα in HEK293 cells with indicated transfections and E2 treatment. *J–K*, immunoblots showing the interaction between SIRT7 and WT-ERα, 2KQ-ERα or 2KR-ERα in HEK293 cells with the indicated treatments.

As previously reported, acetylation of ERα at K302/303 is critical for maintaining its protein stability through reinforcement of the chaperone complex (25). Interestingly, we found that SIRT7 interacted with ERα under ligand-free conditions (Fig. 5, *A* and *B*). Overexpression of SIRT7 markedly reduced ERα protein acetylation, whereas the K302/303R mutant (ERα-2KR) showed minimal change (Fig. 5*C*), indicating that SIRT7 directly deacetylates unliganded ERα at these residues. Supporting this notion, ERα-2KR (a hypoacetylation mimic) more effectively stabilized protein stability of both exogenous and endogenous SIRT7 compared with wild-type ERα (Fig. 5, *D* and *E*) and significantly reduced SIRT7 polyubiquitination (Fig. 5*F*). These findings suggest that deacetylated ERα competes with SIRT7 for binding to STUB1, the E3 ligase responsible for SIRT7 protein degradation under ligand free conditions. We next examined the effect of E2 on the SIRT7-ERα interaction. Upon E2 stimulation, the association between SIRT7 and ERα was markedly diminished (Fig. 5*G*), coinciding with increased ERα protein acetylation (Fig. 5*H*). Consistently, SIRT7-mediated ERα protein deacetylation was largely abolished by E2 treatment (Fig. 5*I*). Notably, E2 disrupted the interaction between SIRT7 and wild-type ERα in a time-dependent manner but had little effect on ERα-2KR (Fig. 5*J*), whereas the ERα-2KQ mutant (a hyperacetylation mimic) exhibited a similar response to wild-type ERα (Fig. 5*K*). Collectively, these results suggest that SIRT7-mediated deacetylation of ERα at K302/303 stabilizes SIRT7 protein and governs its distinct response to E2.

### SIRT7-mediated deacetylation promotes STUB1-mediated degradation of misfolded ERα protein

**Figure 6:**
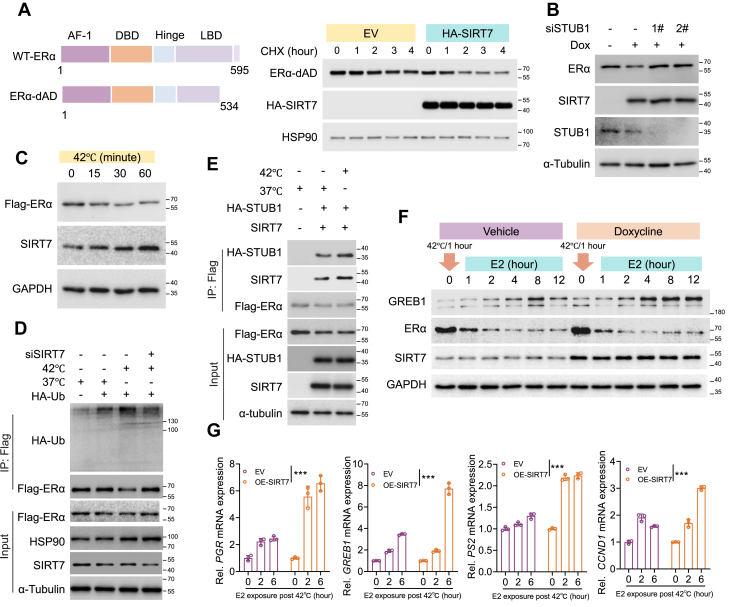
**SIRT7 promotes STUB1-mediated degradation of misfolded Erα.***A*, immunoblots (*right panel*) showing the protein turnover of ERα-dAD (*left panel*, ERα lacking amino acid residues 534–595) with or without SIRT7 overexpression. *B*, immunoblotting analysis of ERα protein level in MCF7 cells with SIRT7 overexpression and/or STUB1 knockdown. Dox, doxycline-induced SIRT7 overexpression, driven by Tet-On system, 0.5 μg/ml. *C*, immunoblotting analysis of ERα and SIRT7 expression in HEK293 cells cultured in 42 °C for indicated time points. *D*, immunoblotting analysis of ERα polyubiquitination in HEK293 cells with indicated transfections and thermal condition. *E*, immunoblots showing the Co-IP elutes of anti-Flag-ERα beads derived from HEK293 cells with indicated transfections and thermal status. *F–G*, immunoblotting (*F*) and qPCR (*G*) analysis of samples from MCF-7 cells with SIRT7 overexpression or not, following incubation at 42 °C for 1 h and subsequent E2 treatment for the indicated durations. Bars, mean ± SD from three independent experiments. The *p* values by two-way ANOVA, ∗∗∗*p* < 0.001.

STUB1 predominantly localizes to the cytoplasm and facilitates the degradation of unliganded or misfolded ERα receptor (24). We therefore asked whether SIRT7 was essential for the protein quality control of ERα. Using an ERα mutant lacking the E2-dependent activation domain (ERα-ΔAD) to exclude ligand effects (26), SIRT7 overexpression markedly accelerated ERα-ΔAD protein turnover (Fig. 6*A*), indicating that SIRT7 participates in ERα protein elimination under ligand-free conditions. Consistently, doxycycline-induced SIRT7 overexpression promoted ERα protein downregulation, whereas STUB1 knockdown abolished this effect (Fig. 6*B*), supporting a cooperative role for SIRT7 and STUB1 in maintaining ERα proteostasis. To further assess this mechanism, we applied thermal stress (42 °C) to induce ERα protein misfolding (24). As shown, heat-stress triggered a pronounced reduction in ERα but an increase in SIRT7 protein expression (Fig. 6*C*). Accordingly, while thermal stress markedly promoted ERα polyubiquitination, this effect was largely abrogated by SIRT7 knockdown (Fig. 6*D*). Supporting the involvement of STUB1, we observed a substantial increase in ERα-STUB1 protein interaction under heat stress (Fig. 6*E*). Functionally, MCF-7 cells subjected to thermal stress coinciding with SIRT7 overexpression exhibited a markedly enhanced responsiveness to E2 stimulation, as evidenced by the higher expression of ERα target genes (Fig. 6, *F* and *G*), implicating that the clearance of misfolded ERα protein is essential for E2-ERα signaling activation. Together, these results identify SIRT7 as a critical factor that cooperates with STUB1 to eliminate misfolded ERα, thereby safeguarding the receptor quality and preserving estrogen signaling fidelity.

### Dysregulation of the SIRT7–ERα axis in disease pathogenesis

**Figure 7:**
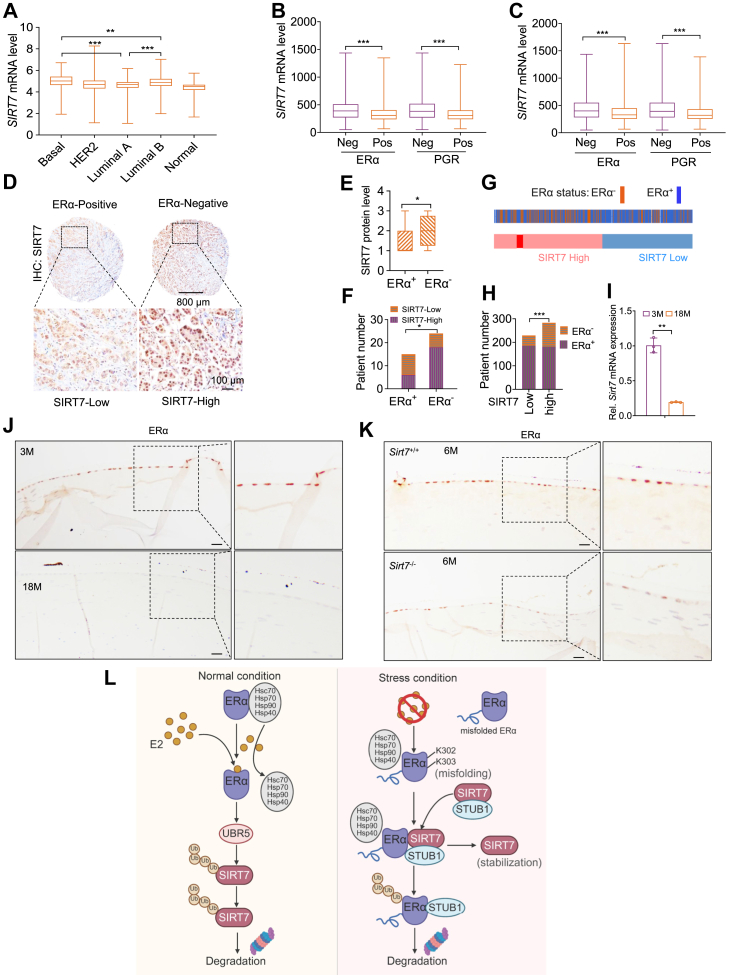
**Dysregulation of the SIRT7-ERα axis in estrogen-related pathologies.***A–C*, Box-and-whisker plots illustrating SIRT7 expression across defined breast cancer subtypes using publicly available datasets; The *p* value by unpaired two-sided Student’s *t* test, ∗∗*p* < 0.01, ∗∗∗*p* < 0.01. *D–F*, representative images (*D*) showing IHC staining of SIRT7 in ERα-positive and ERα-negative breast cancer tissues, with SIRT7 expression levels presented as IHC scores (*E*) and its distribution across 39 patients (F) based on defined standards; The *p* value in (E), unpaired two-sided Student’s *t* test; and in (*F*), Chi-squared test; ∗*p* < 0.05. *G–H*, analysis of ERα status in breast cancer patients categorized by high or low SIRT7 expression based on the public datasets (METABRIC). *I*, qPCR analysis of SIRT7 mRNA expression in young and aged mice; Bars, mean ± SD (n = 3); The *p* value by unpaired two-sided Student’s *t* test, ∗∗*p* < 0.01. *J–K*, representative images showing IHC staining of ERα expression in the lens of young and aged mice (*J*) or in 6-month-old SIRT7 wild-type and knockout mice (*K*); Scale bars, 50 μm. *L*, schematic illustration summarizing the mutual regulation between SIRT7 and ERα, and the cooperative role of SIRT7 and STUB1 in maintaining ERα proteostasis and modulating estrogen signaling (Created in BioRender. Han Y. (2026) https://BioRender.com/9wl63if).

We next investigated the pathological relevance of the reciprocal regulation between SIRT7 and ERα. Using hormone receptor-positive breast cancers as an initial example, we found that *SIRT7* mRNA expression was significantly reduced in luminal A tumors compared with basal-like or luminal B subtypes (Fig. 7*A*). Because luminal A tumors are predominantly ERα-positive, further stratification unraveled the lower SIRT7 expression in ERα^+^ and PGR^+^ cases relative to receptor-negative tumors (Fig. 7, *B* and *C*). Consistently, immunohistochemical staining analysis of a breast cancer tissue microarray showed an inverse correlation between SIRT7 and ERα protein expression (Fig. 7, *D*–*F*). Notably, loss of ERα was more frequently observed in tumors with elevated SIRT7 expression (Fig. 7, *G* and *H*), suggesting disruption of the SIRT7–ERα regulatory equilibrium in hormone-dependent breast cancer, which may contribute to endocrine resistance. Given that SIRT7 expression declines during aging and cellular senescence (27), and that estrogen signaling has been implicated as a critical factor contributing to age-related cataract (ARC) formation among postmenopausal women (28), we further examined this regulatory axis in lens aging. Compared with young mice, both SIRT7 and ERα levels were markedly reduced in aged lenses (Fig. 7, *I* and *J*). Consistently, SIRT7 knockout led to an apparent downregulation of ERα protein expression in lens epithelial cells (Fig. 7*K*), although no overt cataract formation was observed within the limited observation period. Collectively, these findings suggest that dysregulation of the SIRT7–ERα axis is associated with pathological contexts such as breast cancer and aging-related diseases.

## Discussion

Emerging evidence has established the critical roles of SIRT7 in diverse physiological and pathological processes. Regarding sexual dimorphism, several studies have revealed intriguing but mechanistically unresolved connections. In this study, we demonstrate that the reciprocal regulation between ERα and SIRT7 converges on STUB1-mediated proteasomal degradation. Under ligand-free conditions, when ERα is properly folded and assembled into a stable native complex, STUB1 preferentially targets SIRT7 for degradation. In contrast, under pathological or stress conditions that promote the accumulation of misfolded ERα, SIRT7 binds tightly to ERα and deacetylates it at K302/303. This deacetylation enhances their interaction and facilitates STUB1-mediated recognition and degradation of the misfolded receptor, thereby enforcing ERα protein quality control and preserving normal estrogen signaling. Importantly, upon estrogen stimulation, E2-bound ERα activates non-genomic MAPK signaling, which induces UBR5-mediated SIRT7 degradation, establishing a feedback loop that fine-tunes estrogen receptor activity. A schematic diagram summarizing this mechanism is presented in Figure 7*L*. Given that estrogen and its receptors are key determinants of sexual dimorphism, our findings position SIRT7 as a critical regulator of hormonal homeostasis and sexual dimorphism. Interestingly, SIRT7 deficiency extends lifespan in male mice (29), whereas female Sirt7 knockout mice exhibit reduced fecundity characterized by premature fertility decline and depletion of the oocyte pool. In particular, SIRT7 has been shown to be essential for establishing the ovarian reserve and maintaining reproductive longevity in females, as its loss leads to reduced numbers of primordial follicles and early-onset infertility (30). Together, current study uncovers a molecular mechanism linking SIRT7 to ERα regulation and signaling fidelity. Future studies employing appropriate animal models, particularly those relevant to aging and hormone-dependent cancers, will be essential to delineate these functions *in vivo*.

The chaperone-associated E3 ligase STUB1 (CHIP) forms complexes with Hsp90, Hsp70, Hsp40, and BAG-1 to target unliganded ERα for ubiquitination and degradation (24, 25, 31). Our findings further reveal that SIRT7 facilitates STUB1 recognition of unliganded or misfolded ERα, promoting its ubiquitination and proteasomal clearance. These results suggest that SIRT7 functions as a quality-control deacetylase, coupling deacetylation to the selective ubiquitination of non-native client proteins within a chaperone-assisted complex. Supporting this notion, previous studies have shown that SIRT7 knockout markedly upregulates ATF4 (29), a principal transcriptional effector of the unfolded protein response (UPR), and participates into the regulation of ER stress (5, 29, 32, 33), highlighting a mechanistic link between SIRT7 activity and cellular proteostasis. Given the broad range of nuclear receptor substrates recognized by STUB1, it is plausible that SIRT7 participates in the regulation of multiple nuclear receptors. Indeed, emerging evidence indicates that SIRT7 interacts with and modulates the activity of PPARγ2 (34) and TR4 (6). Whether additional nuclear receptors are subject to SIRT7/STUB1-mediated protein quality control warrants systematic investigation in future studies.

Although SIRT7 was initially identified as a nucleolar protein, it is known to shuttle among the nucleolus, nucleoplasm, and cytoplasm. The cytoplasmic functions of SIRT7 remain less well characterized, yet several studies suggest its involvement in regulating signaling pathways such as EGFR and AKT (3, 35). Notably, STUB1 is predominantly localized in the cytoplasm and the degradation of misfolded ERα has also been reported to occur within this compartment (24). These observations imply that SIRT7-mediated ERα ubiquitination primarily takes place in the cytoplasm, despite its relatively low abundance in this fraction as observed in the current study. However, we cannot exclude the possibility of cytoplasmic accumulation of SIRT7 under prolonged stress conditions associated with systemic changes, such as aging, unfolded protein response (UPR) activation, and tumorigenesis. Our findings thus suggest that the cytoplasmic pool of SIRT7 may play a pivotal role in maintaining protein quality control, thereby linking its cytoplasmic function to multiple pathological contexts and disease processes.

In summary, this study uncovers an interesting role of SIRT7 in safeguarding ERα proteostasis through deacetylation-dependent degradation of unliganded and misfolded receptors, while simultaneously modulating its own protein turnover *via* estrogen signaling or E3 ligase competition. The differential turnover kinetics of ERα and SIRT7 in this model are largely determined by (1): ligand-dependent degradation of ERα *versus* signaling-driven degradation of SIRT7 (2); reciprocal competition between SIRT7 and ERα for STUB1 binding under ligand-free conditions; and (3) spatially distinct actions of UBR5 and STUB1 on nuclear *versus* cytoplasmic protein pools. Future studies employing advanced techniques to monitor protein degradation across subcellular compartments could further clarify this spatial regulation and deepen our mechanistic understanding of SIRT7’s role in hormonal homeostasis and related diseases.

## Experimental procedures

### Cell culture

HEK293T, T47D, and MCF-7 cells were cultured in high-glucose DMEM (PM150210, Procell), whereas MDA-MB-231 cells were maintained in MEM (PM150410, Procell). All media were supplemented with 10% fetal bovine serum (FBS; BC-SE-FBS07, Nanjing SenBeiJia Biological Technology) and 1% penicillin/streptomycin (BC-CE-007, Nanjing SenBeiJia Biological Technology). All cell lines were obtained from the American Type Culture Collection (ATCC) and authenticated by morphology and growth characteristics. Routine *mycoplasma* testing was performed using PCR-based assays (Takara, #6601).

### Transfection and lentivirus production

Transient transfections of plasmids or siRNAs were performed using Lipofectamine 3000 (L3000015, Thermo Fisher Scientific) or polyethyleneimine (PEI; 40816ES02, Yeasen, China) according to the manufacturers’ instructions. All primers, shRNAs, and siRNAs were synthesized by Sangon Biotech or GenePharma . For lentivirus production, HEK293T cells grown in 10-cm dishes were co-transfected with the respective lentiviral expression plasmids and packaging vectors pMD2.G and psPAX2 at a ratio of 1:0.5:1. Viral supernatants were collected, filtered, and used to infect target cells in the presence of polybrene (4 μg/ml). After 12 h, infected cells were replaced with fresh medium. At 48 h post-infection, cells were selected using puromycin (0.25–1 μg/ml) or sorted by fluorescence-activated cell sorting (FACS). For Tet-On system, the lentiviral vector pLVX-TRE3G-IRES (Takara) was used, and gene expression was induced by doxycycline treatment (HY-N0565, MCE).

### RNA extraction and quantitative PCR analysis (qPCR)

Total RNA was isolated from cells based on the protocol using TRIzol reagent (Takara). For cDNA synthesis, 500 ng of RNA was reverse-transcribed using the PrimeScript RT reagent kit (Takara, RR014). Quantitative PCR was conducted using the SYBR Green method (Takara, RR064) on a real-time PCR detection system. Gene expression levels were calculated using the 2^-ΔΔCt^ method after normalization to the relevant internal control. Primer sequences are listed in Table S2.

### Plasmid constructs

Plasmids were generated using PCR amplification (YEASEN, 10154ES, China) followed by cloning with the One Step Cloning Kit (Vazyme Biotech, C112) into the pcDNA3.1 vector (Thermo Fisher Scientific) or the pLVX-IRES-ZsGreen1 and pLVX-TRE3G-IRES lentiviral vector (Takara). All constructs were verified by DNA sequencing (Beijing Tsingke Biotech). Primers sequences used in this study are provided in Table S2.

### Protein extraction, immunoprecipitation, and immunoblotting

For co-immunoprecipitation (Co-IP), cells were lysed in IP lysis buffer (25 mM Tris-HCl pH 7.4, 150 mM NaCl, 1% NP-40, 1 mM EDTA) supplemented with protease and phosphatase inhibitor cocktails (Sangon Biotech). Clarified lysates were incubated with the indicated primary antibodies for 2 h at 4 °C, followed by an additional 4 h or overnight incubation with protein A/G beads. After sufficient washing with lysis buffer, bound proteins were eluted by boiling in SDS sample buffer and subjected to immunoblotting. For details, proteins were separated by SDS-PAGE and transferred to PVDF membranes. Membranes were blocked and probed with the indicated primary and HRP-conjugated secondary antibodies, and immunoblots were captured using the BG-gdsAUTO 710 imaging system (Baygene Biotech). Band intensities were quantified using Image J software (v1.52). Details of all antibodies are provided in Table S1.

### *In vivo* ubiquitination assay

HEK293T, MCF-7 or MDA-MB-231 cells were transfected into indicated plasmids or siRNAs for 48 h. Following appropriate treatments, cell lysates were prepared in immunoprecipitation lysis buffer (IP buffer, 50 mM Tris-HCl pH 7.4, 150 mM NaCl, 1% NP-40, 0.5% deoxycholic acid, 0.1% SDS, 10 mM NaF, 1 mM dithiothreitol), supplemented with protease and phosphatase inhibitors (Sangon Biotech). Cell lysates were then centrifuged to obtain clear supernatants, which were then incubated with the indicated primary antibodies and agarose beads for immunoprecipitation. Post-washing by the lysis buffer to remove non-specifically bound proteins, immunoprecipitates were eluted in SDS sample buffer and analyzed by immunoblotting to detect ubiquitinated proteins.

### Animals

*Sirt7*^−/+^ mice were obtained as previously described (23, 36). All animal experiments were performed in accordance with protocols approved by the Committee on the Use of Live Animals in Teaching and Research at Hunan University (Approval Number: HNU-IACUC-2023-103).

### Immunohistochemical analysis (IHC)

SIRT7 expression in human breast cancer tissue samples and ERα expression in mouse lenses were evaluated by immunohistochemical (IHC) staining following the manufacturer’s standard protocol (SP0041, Solarbio, China). Human breast cancer tissue microarrays (BR963b) with defined pathological information were obtained from Alena Bio (Xi’an). Staining scores were determined based on the percentage of positive tumor cells and staining intensity: 0 = negative, 1 = low, 2 = moderate, and 3 = high expression. For statistical analysis of the association between SIRT7 and ERα status using the Chi-squared test, samples were stratified as low SIRT7 expression (scores 0–1) or high SIRT7 expression (scores 2–3).

### Statistical analysis

All statistical analyses were performed using GraphPad Prism software (Version 8.0, GraphPad Software). Represented data were obtained from at least three independent biological replicates. Results are presented as the mean ± SD. Comparisons between two groups were analyzed using a two-tailed Student’s *t* test, while multiple-group comparisons were analyzed using two-way ANOVA. The Chi-squared test was applied to analyze categorical variables. Statistical significance was defined as follows: ∗*p* < 0.05; ∗∗*p* < 0.01; ∗∗∗*p* < 0.001; and n.s. (no significance) for *p* > 0.05.

## Data availability

Datasets used for the analyses in Figure 7 were obtained as follows: Figure 7*A*, GSE202203; Figure 7*B*, TCGA (Cell, 2015) (37), and Figure 7*C*, TCGA (*Firehose Legacy*), both accessed *via* the cBioPortal platform; and Figure 7, *G* and *H*, the METABRIC cohort (38, 39). All data supporting the findings of this study are available from the corresponding author upon reasonable request.

## Supporting information

This article contains supporting information (Table S1 and S2).

## Conflict of interest

The authors declare that they have no conflicts of interest with the contents of this article.
