# Supplementary material for: SIRT7 safeguards ERα proteostasis via deacetylation-dependent degradation of unliganded and misfolded receptors
Source: J Biol Chem. 2026 Jan 20;302(3):111173. doi: 10.1016/j.jbc.2026.111173 (PMC12919253; doi:10.1016/j.jbc.2026.111173)
Supplement: Supporting Tables [file mmc1.pdf]

SIRT7 safeguards ERα proteostasis via deacetylation-dependent degradation of unliganded and misfolded receptors

Supplementary Information

Supplementary Table 1. List of antibodies used in this study

Antibody	Source	Dilutions (WB, Western blotting; IP, immunoprecipitation)
SIRT7	Santa Cruz (sc-365344)	WB/IP (1:3,000/1:100)
SIRT7	EMD Millipore (ABE103)	IHC (1:50)
HSP90	Abcam (ab13495)	WB (1:3,000)
GREB1	Proteintech (28699-1-AP)	WB (1:3,000)
PGR	Proteintech (25871-1-AP)	WB (1:3,000)
ERα	Thermo Fisher (MA5-13191)	WB (1:3,000)
Pan-acetylation	PTM BIO (PTM-105)	WB (1:2,000)
STUB1	Santa Cruz (sc-133066)	WB (1:1,000)
HA-tag	Sigma–Aldrich (H3663)	WB (1:5,000)
FLAG-tag	Sigma–Aldrich (F3165)	WB (1:5,000)
α-Tubulin	Beyotime (AT819)	WB (1:5,000)
GAPDH	Beyotime (AG019)	WB (1:5,000)
Anti-mouse IgG	Jackson (11-035-003)	WB (1:10,000)
Anti-rabbit IgG	Jackson (15-035-003)	WB (1:10,000)
EDD1/UBR5	Abcam (ab70311)	IP (1:500)
Anti-FLAG Affinity Gel	Yeasen (20584ES)	IP

**Supplementary Table 2. List of oligonucleotides used in this study**		
**Targets**	**Sequence (5'-3')**	**Purpose**
h*SIRT7*-1#	CUCACCGUAUUUCUACUACUAdTdT	siRNA
h*SIRT7*-2#	CACCUUUCUGUGAGAACGGAAdTdT	siRNA
h*EDD1*-1#	AACUUAGAUCUCCUGAA	siRNA
hEDD1-2#	AGACAAAUCUCGGACUUGA	siRNA
h*STUB1*-1#	CCAAGCACGACAAGUACAUdTdT	siRNA
h*STUB1*-2#	GGAGCAGGGCAAUCGUCUGdTdT	siRNA
h*ERα*-1#	UUUGCAAGGAAUGCGAUGAdTdT	siRNA
h*ERα*-2#	GGAGAAUGUUGAAACACAAdTdT	siRNA
h*ESR1*-F	ATCCTGATGATTGGTCTCGTCT	qPCR
h*ESR1*-R	GGATATGGTCCTTCTCTTCCAGA	qPCR
h*pS2*-F	CCAGTGTGCAAATAAGGGCTGC	qPCR
h*pS2*-R	AGGCAGATCCCTGCAGAAGTGT	qPCR
h*β-Actin*-F	AGAGCTAGCTGCCTGAC	qPCR
h*β-Actin*-R	GGATGCCACAGGACTCCA	qPCR
h*SIRT7*-F	ATGAGCAGAAGCTGGTGC	qPCR
h*SIRT7*-R	CTGTCTGGTGTCTGTGGA	qPCR
h*GREB1*-F	GGTCTGCCTTGCATCCTGATCT	qPCR
h*GREB1*-R	TCCTGCTCCAAGGCTGTTCTCA	qPCR
h*PgR*-F	GTCGCCTTAGAAAGTGCTGTCAG	qPCR
h*PgR*-R	GCTTGGCTTTCATTTGGAACGCC	qPCR
sh-hSIRT7-1	CCGGGCCTGAAGGTTCTAAAGAACTCGA GTTCTTTAGAACCTTCAGGCTTTTT	shRNA
sh-hSIRT7-2	CCGGGAACGGAACTCGGGTTATTCTCGAGAA TAACCCGAGTTCCGTTCTTTTT	shRNA
sh-hESR1-1	CCGGTTTGCAAGGAATGCGATGACTCGA GTCATCGCATTCCTTGCAAATTTTT	shRNA
sh-hESR1-2	CCGGGGAGAATGTTGAAACACAACTCG AGTTGTGTTTCAACATTCTCCTTTTT	shRNA
